# Tribbles Homolog 3-Mediated Vascular Insulin Resistance Contributes to Hypoxic Pulmonary Hypertension in Intermittent Hypoxia Rat Model

**DOI:** 10.3389/fphys.2020.542146

**Published:** 2020-10-30

**Authors:** Fang Fan, Jinxiao He, Hui Su, Haifeng Zhang, Hao Wang, Qianqian Dong, Minghua Zeng, Wenjuan Xing, Xin Sun

**Affiliations:** ^1^Department of Pediatrics, Xijing Hospital, Fourth Military Medical University, Xi’an, China; ^2^Department of Geratology, Xijing Hospital, Fourth Military Medical University, Xi’an, China; ^3^Teaching Experiment Center, Fourth Military Medical University, Xi’an, China; ^4^Department of Natural Medicine, School of Pharmacy, Fourth Military Medical University, Xi’an, China; ^5^School of Aerospace Medicine, Fourth Military Medical University, Xi’an, China; ^6^State Key Laboratory of Space Medicine Fundamentals and Application, China Astronaut Research and Training Center, Beijing, China

**Keywords:** hypoxic pulmonary hypertension, proliferator-activated receptor gamma, pulmonary arterial endothelium, vascular insulin resistance, Tribbles homolog 3

## Abstract

This study aimed to investigate the role of vascular insulin resistance (VIR) and Tribbles homolog 3 (TRIB3) in the pathogenesis of hypoxia-induced pulmonary hypertension (HPH). Rats were subjected to low air pressure and low oxygen intermittently for 4 weeks to induce HPH. The mean right ventricular pressure (mRVP), mean pulmonary arterial pressure (mPAP), and right ventricular index (RVI) were significantly increased in HPH rats. Pulmonary arteries from HPH rats showed VIR with reduced vasodilating effect of insulin. The protein levels of peroxisome proliferator-activated receptor gamma (PPARγ), phosphoinositide 3-kinase (PI3K), phosphorylations of Akt, and endothelial nitric oxide (NO) synthase (eNOS) were decreased, and TRIB3 and phosphorylated extracellular signal-regulated protein kinases (ERK1/2) were increased in pulmonary arteries of HPH rats. Early treatment of pioglitazone (PIO) partially reversed the development of HPH, improved insulin-induced vasodilation, and alleviated the imbalance of the insulin signaling. The overexpression of TRIB3 in rat pulmonary arterial endothelial cells (PAECs) reduced the levels of PPARγ, PI3K, phosphorylated Akt (p-Akt), and phosphorylated eNOS (p-eNOS) and increased p-ERK1/2 and the synthesis of endothelin-1 (ET-1), which were further intensified under hypoxic conditions. Moreover, TRIB3 knockdown caused significant improvement in Akt and eNOS phosphorylations and, otherwise, a reduction of ERK1/2 activation in PAECs after hypoxia. In conclusion, impaired insulin-induced pulmonary vasodilation and the imbalance of insulin-induced signaling mediated by TRIB3 upregulation in the endothelium contribute to the development of HPH. Early PIO treatment improves vascular insulin sensitivity that may help to limit the progression of hypoxic pulmonary hypertension.

## Introduction

Pulmonary hypertension (PH) is a progressive disease featuring vascular remodeling of small pulmonary arteries, elevated pulmonary arterial resistance, and right ventricle hypertrophy (Latus et al., 2015). The mortality and morbidity of PH are remarkable. The etiology of PH is very complicated; it can be idiopathic, heritable, drug/toxin-induced, or associated with HIV infection, congenital heart diseases, lung diseases, and high-altitude habitancy. The development of pulmonary circulation disorders in hypoxia-induced pulmonary hypertension (HPH) is a response unique to the hypoxic environment. This pathological condition triggers the hypoxic vasoconstriction of pulmonary arteries to redistribute the blood flow from hypoxic alveoli to ample alveoli so as to fully oxygenate the blood (Serne et al., 1999; Kekalainen et al., 2000). In the long term, this compensatory strategy can intensify the constriction of pulmonary vessels and eventually cause endothelial dysfunction, remodeling, and stenosis of vessels. Therefore, acute and chronic hypoxia is one of the most effective and reproducible method to induce PH.

Insulin is a key regulator of blood glucose in the body. The condition of insulin resistance is a precursor to developing type 2 diabetes and many cardiovascular disorders. Insulin also has a vasoactive effect on the vascular endothelium in parallel with the metabolic effect on tissues (Munir and Quon, 2013; Xing et al., 2013a,b). It stimulates the production and release of nitric oxide (NO) from the vascular endothelial cells through protein kinase B (also known as Akt) mediated phosphorylation of endothelial NO synthase (eNOS) in a phosphoinositide 3-kinases (PI3K) dependent manner (Yu et al., 2011). Simultaneously, insulin triggers the activation of the mitogen-activated protein kinase (MAPK) pathway to promote the secretion of endothelin-1 (ET-1). The overall response of insulin on the healthy vascular endothelium depends on a balance of the two pathways and presents a vasodilated effect (Muniyappa et al., 2007). Abated vasodilatory effects and augmented vasoconstrictor actions of insulin are referred to as vascular insulin resistance (VIR). The molecular basis of VIR is selectively impaired in insulin-induced PI3K-dependent and intact or even enhanced MAPK-dependent signaling pathways (Muniyappa et al., 2020). The underproduction of NO and the imbalance of NO/ET-1 production are the signposts of endothelial dysfunction. VIR and endothelial dysfunction are vicious cycles in the development of diabetes, cardiovascular disease, and hypertension. However, whether VIR participated in the development of pulmonary circulation disorders and HPH remains largely elusive.

Tribbles homolog 3 (TRIB3) is a pseudokinase of the Tribbles family (Du et al., 2003). It is involved in various cellular functions, including endoplasmic reticulum stress, cell proliferation, and differentiation. TRIB3 is coupled to insulin resistance in oxidative stress, antioxidant stress, inflammation, adiponectin actions, peroxisome proliferator-activated receptor (PPAR) actions, SIRT1 actions, and insulin signal transduction (Koh et al., 2013; Zhang et al., 2016). Overexpression of TRIB3 downregulates insulin-induced Akt phosphorylation, and subsequent downstream insulin signaling and suppression of TRIB3 enhances insulin-stimulated signaling. Several studies demonstrated the key role of TRIB3 in regulating insulin’s metabolic function. High-fat diet induced the high TRIB3 expression, which consequently affects insulin-induced glucose uptake and oxidation in muscle and fat tissues in rats (Liu et al., 2012a). TRIB3 proteins are upregulated in diabetic rats and distorted phosphorylation of Akt, and MAPK in diabetic rats is restored by the suppression of TRIB3 (Ti et al., 2011). Hypoxia was reported that induced TRIB3 expression (Wennemers et al., 2011). However, whether TRIB3 is responsible for VIR in HPH is still unknown.

Life style like exercise and intervention such as PPAR gamma (PPARγ) agonist pioglitazone (PIO) have been shown to improve cardiovascular insulin sensitivity and benefit cardiovascular function (Zhang et al., 2008). Most recently, PIO has been reported to reverse pulmonary arterial hypertension, another type of PH, and reverse vascular remodeling *via* fatty acid oxidation (Legchenko et al., 2018), inhibiting transforming growth factor-β (TGFβ1) signaling (Calvier et al., 2019) and 5-HT receptor signaling (Liu et al., 2012b). In addition, systemic insulin resistance (i.e., elevated fasting blood glucose and insulin levels) is associated with the development of pulmonary arterial hypertension (Hansmann et al., 2007) and treatment with PIO abnormal pulmonary artery muscularization. Whether PIO limited VIR in the development of hypoxia-induced pulmonary arterial disorders and HPH is still unknown. This study aimed to investigate the alterations and the mechanisms of the vascular insulin sensitivity of pulmonary arteries in an HPH rat model and the potential role of PIO in HPH.

## Materials and Methods

### Animals

The 6-week male Sprague-Dawley rats (body weight: 200–220 g) were purchased from the Experimental Animal Center of the Fourth Military Medical University (Xi’an, Shaanxi province, China). They were fed with normal diet and water *ad libitum*. The experimental protocol was approved by the ethics committee of the Animal Experimentation of the Fourth Military Medical University and conducted according to the Guidelines of the Animal Experimentation of the Fourth Military Medical University.

### Design of the Experiment

The rats were randomly assigned to four groups with seven rats in each group: normal control, HPH, HPH + PIO (E), and HPH + PIO (L). The rats in the last three groups were subjected to low air pressure (50 kPa) and low oxygen (10%) intermittently in an auto-modulating plastic cabin for 8 h and normoxia for 16 h every day, for a total of 4 weeks (Li et al., 2020). Anhydrous calcium chloride and soda lime were used in the plastic box to absorb water and CO_2_. PIO pellets were dissolved in physiological saline to form a solution (1.5 mg/ml). Besides, HPH + PIO (E) group rats received early (E) PIO solution [10 mg/(kg d)] once daily for 7 days a week through intragastric administration in the 2nd week of the experiment. HPH + PIO (L) group rats received late (L) treatment of PIO in the same way in the last week of the experiment. HPH group rats were given saline as control. The normal control group was maintained in the same room under normal conditions (at atmospheric pressure and oxygen concentration).

### Hemodynamic Measurement and Right Ventricular Hypertrophy

The mean pulmonary arterial pressure (mPAP) and mean right ventricular pressure (mRVP) were measured by jugular vein catheterization and followed the methodology in the previous study (Michelakis et al., 2002).

In addition, the right ventricular index (RVI) was calculated as an indicator of right ventricular hypertrophy. RVI represents the weight of the RV relative to LV + septum.

### Morphometric Analysis

The same full section in the mid-portion of the lung parallel to the hilum was taken from all rats and embedded in paraffin. Transverse 10-μm left lung sections were stained using Verhoeff-van Gieson staining and H&E staining. The external diameter (ED), medial wall thickness (MT), medial cross-sectional area (MA), and total arterial cross-sectional area (TAA) of the peripheral pulmonary artery were measured in micrographs. Then, the MT (%) and MA (%) were calculated as follows: MT% = (MT/ED) × 100% and MA% = (MA/TAA) × 100%. The two ratios indicated the level of pathological thickening and remodeling of pulmonary arteries. Further, 7–10 pulmonary arteries were picked from random views of every lung, and the averages of morphometric parameters were used for conducting the statistical analysis.

### Isometric Tension Measurement

The pulmonary arteries were isolated from rats and cut into 3-mm rings. Vascular responses to insulin in arteries were studied by exposing pulmonary arterial segments to insulin (10^−10^ ~ 10^−6^ mol/L; Sigma-Aldrich, MO, United States), sodium nitroprusside (SNP) (10^−10^~10^−6^ mol/L; Sigma-Aldrich, MO, United States), or PE (10^−10^~10^−5^ mol/L; Sigma-Aldrich, MO, United States). PE (10^−7^ mol/L) was used before testing relaxation to insulin and SNP. The resulting changes in isometric tension were recorded using an RM6240 multichannel physiological recording and processing system (Chengdu Instrument Factory, China).

### Primary Culture of Rat Pulmonary Microvascular Endothelial Cells

Rat primary pulmonary microvascular endothelial cells were cultured and maintained as previously described with modifications. The rats were anesthetized by the intraperitoneal injection of 0.3–0.5 ml/100 g chloral hydrate. The thoracic and abdominal regions were sterilized by applying 1% povidone-iodine on the skin. The abdominal cavity was opened, and the abdominal aorta was cut off to drain the blood. Subsequently, the thoracic cavity was opened, and the lungs were immediately removed and rinsed in phosphate-buffered saline (PBS) at pH equal to 7.4 thoroughly. About 1 mm of the fringe of the lung was cut off and cut into pieces of 0.5 × 0.5 × 0.5 mm^3^ in fetal bovine serum (FBS). Small pieces of lung tissue were placed evenly at the bottom of the cell culture flask. The flask was placed upside down for incubation at 37°C (210 ml/L O_2_) with minimal cell culture medium (M200; Sigma-Aldrich; 15% FBS and 20 g/L LSGS) at the bottom. The flask was reverted back after 4 h. The medium was replaced after 24 h to wash off erythrocytes. Small pieces of lung tissues were removed after 2 days of incubation. Light microscopic images showed the cobblestone morphology of cultured cells typical for endothelial cells. Factor VIII was positive in immunofluorescence staining. The cells were split after >80% confluence at the ratio of 1:2 and used for experiments between passages 2 and 4. A rabbit anti-human factor VIII antibody was purchased from Santa Cruz Biotechnology (TX, United States). Fluorescein isothiocyanate (FITC)-conjugated goat anti-rabbit immunoglobulin G was purchased from Abcam (Cambridge, United Kingdom).

### *In vitro* Experiment Design

Experimental design No. 1: healthy pulmonary arterial endothelial cells (PAECs) at passages 2–4 were plated in a 60-mm cell culture dish (Corning) in 10% FBS M200 medium. After the cells grew to 60% confluence, a fresh medium with 10^−7^ mmol/L insulin was replaced. The dishes were then divided into normoxia and hypoxia groups and placed in a normoxia incubator (37°C, 210 ml/L O_2_) and a hypoxia incubator (37°C, 100 ml/L O_2_), respectively. The cells and culture media were collected after 6, 24, 48, and 96 h of incubation.

Experimental design No. 2: healthy PAECs at passages 2–4 were plated in a 60-mm cell culture dish (Corning) in 10% FBS M200 medium. For exposure to hypoxia, PAECs were placed in a hypoxia incubator (Thermo Fisher, Boston, MA, United States) filled with a mixture of 10% O2 and 5% CO2 at 37°C. The normoxic control group was filled with a mixture of 21% O2 and 5% CO2 at 37°C. Cells were treated with 10^−7^ mmol/L insulin. The cells were harvested after 48 h of incubation. The proteins were extracted for the immunoblotting test.

### Small Interfering RNA Transfection

For gene silencing assay, small interfering RNA (siRNA) for TRIB3 messenger RNA (mRNA) was designed and purchased from GenePharma (ShangHai, China). The target sequence used for TRIB3 siRNA knockdown was 5'-GAAGAAACCGUUGGAGUUTT-3'. PAECs were transfected with TRIB3 siRNA or scramble control by Lipofectamine™ RNAiMAX (Invitrogen) following the manufacturer’s instructions. Cells were harvested for western blot analyses 48 h later.

### Quantification of NO and ET-1 Expression

The NO concentration in the cell culture media was quantified using a commercial NO assay kit (nitrate reductase method) from Nanjing Jiancheng Bioengineering Institute (Jiangsu Province, China). Total RNA was isolated from cells using TRIzol reagent according to the manufacturer’s protocol (TaKaRa, Japan). Total RNA (0.5 μg) was converted into first-strand complementary DNA in a 20-μl reaction mixture using a One-step Reverse Transcriptase kit (TaKaRa). ET-1 expression was determined by real-time quantitative PCR (forward: 5'-CAAACCGATGTCCTCGTA-3' and reverse: 5'-ACCAAACACATTTCCCTATT-3'). The quantitative expression of the genes was normalized against glyceraldehyde 3-phosphate dehydrogenase (GAPDH; forward: 5'-GATTTGGCCGTATCGGAC-3' and reverse: 5'-GAAGACGCCAGTAGACTC-3'). The reactions were carried out using SYBR Green as a fluorescence dye on a real-time PCR thermal cycler (CFX96 Real-Time PCR cycler; Bio-Rad Laboratories, CA, United States). The 2^−△△^CT method was used for relative expression analysis. The experiment was repeated three times.

### Western Blot Analysis

To analyze the insulin-induced signaling pathway, mice were given hypodermic injections of 5 mU/g body weight insulin (Novo Nordisk, Plainsboro, NJ, United States) and sacrificed 30 min later to collected vascular tissues. Tissues or cells were lysed with western radio immunoprecipitation assay (RIPA) lysis buffer (Beyotime Institute of Biotechnology, China) supplemented with Complete Mini Protease Inhibitor mixture (Roche Applied Science, Penzberg, Germany) and 1% phosphatase inhibitor mixture (Roche Applied Science). The protein concentration was determined using a bicinchoninic acid (BCA) Protein Quantification Kit (Beyotime Institute of Biotechnology, China). Each lysate containing an equal amount of total protein was loaded and separated using the sodium dodecyl sulfate-polyacrylamide gel electrophoresis method, transferred to polyvinylidene fluoride (Invitrogen, CA, United States) membrane, and probed with specific antibodies. After incubation with horseradish peroxidase-conjugated secondary antibodies (Boster Biological Technology, CA, United States), the blots were visualized with enhanced chemiluminescence detection reagents (Millipore, MA, United States), followed by autoradiography using a Kodak developing system. The intensity of images was quantified using Quantity One 4.0. Western blot analysis was performed using antibodies against Akt (60 kDa, 1:1000), phosphorylated Akt (p-Akt; 60 kDa, 1:1000), extracellular signal-regulated protein kinases 1 and 2 (ERK1/2, 42 and 44 kDa, 1:1000), p-ERK1/2 (42 and 44 kDa, 1:1000), and PI3K (110 kDa, 1:1000) obtained from Cell Signaling Technology (MA, United States); phosphorylated eNOS (p-eNOS; 130 kDa, 1:1000) and eNOS (130 kDa, 1:1000) from BD Biosciences (CA, United States); and TRIB3 (45 kDa, 1:200) and PPARγ (55 kDa, 1:500) from Santa Cruz Biotechnology.

### Overexpression of TRIB3 With Lentivirus

Primary PAECs at the second passage were plated into six-well plates. They were infected with the lentivirus harboring *TRIB3* (GeneChem, Shanghai, China) gene according to the manufacturer’s protocol. PAECs (80%) were positively infected with lentivirus (green fluorescence) after 3 days of incubation. The cell culture medium was replaced with fresh M200 medium (Gibco, Invitrogen, CA, United States) with 10% FBS (Hyclone, South Logan, UT, United States) and insulin (10^−7^ mmol/L).

### Statistical Analysis

All values are presented as means ± SEM of *n* independent experiments. Statistical significance was determined by Student’s *t*-test (analysis of two groups) or ANOVA (analysis of four groups), and *post hoc* comparisons, adjusted for multiple comparisons by Bonferroni’s correction, were performed if ANOVAs revealed significances. Repetitive measure ANOVA was used in analyzing vascular tension at different points. In all statistical comparisons, probabilities of 0.05 or less were considered to be statistically significant.

## Results

### Chronic Hypoxia Induced Pulmonary Hypertension and Impaired Insulin-Induced Vasodilation in Pulmonary Arteries

**Figure 1:**
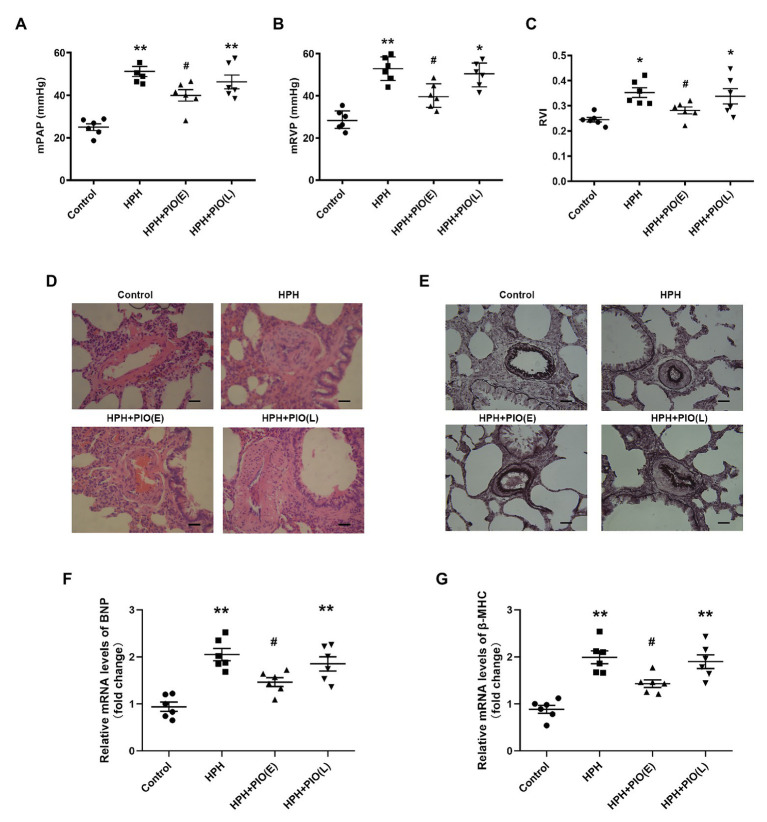
Early treatment with peroxisome proliferator-activated receptor gamma (PPARγ) agonist pioglitazone (PIO) partly reversed the hypoxia-induced pulmonary hypertension (HPH). **(A)** Mean pulmonary artery pressure (mPAP) of different study groups; **(B)** Mean right ventricular pressure (mRVP) of different study groups; **(C)** Right ventricle index (RVI) of different study group. **(D)** Representative microphotographs of pulmonary arteries in rats obtained from four study groups (H&E staining), scale bar = 25 μm; **(E)** Representative microphotographs of pulmonary arteries in rats obtained from four study groups (Verhoeff-van Gieson staining), scale bar = 25 μm. **(F)** and **(G)** messenger RNA (mRNA) levels of brain natriuretic peptide (BNP) and β-myosin heavy chain (β-MHC) in right ventricular of hearts. Data were presented as means ± SEM. *n* = 6–8. ^*^p < 0.05, ^**^p < 0.01 vs. control group; ^#^*p* < 0.05 vs. HPH group.

Chronic hypoxia was induced by exposing animals in HPH group to both hypobaric pressure and oxygen-poor air for 4 weeks. The pathological features of pulmonary vascular function and hypertrophy mimic the ones observed in human PH. The main parameters to define PH were tested in all the studied groups. The mPAP, mRVP, and RVI all significantly increased in rats in the HPH group compared with the control group (*p* < 0.01). Vascular remodeling is an important pathological feature of PH, which leads to increased pulmonary vascular resistance. The histological structure and thickness of pulmonary arteries from the control group were normal. Remodeling of the arteries in the HPH group was manifested by a significant alteration in the ratio of MT to ED (MT%) and the ratio of MA to TAA (MA%; Table 1). The media thickened, and the arterial lumen was narrowed in the HPH group compared with the control group (Figures 1D,E). We also detected RV mRNA levels of brain natriuretic peptide (BNP) and β-myosin heavy chain (β-MHC). Chronic hypoxia caused increased BNP and β-MHC levels (Figures 1F,G), indicating right ventricular hypertrophy.

**Table 1:** Comparing MT and MA% between different study groups (*n* = 6, *x̄* ± *s*).

Group	MT%	MA%
Control	11.0 ± 1.4	25.6 ± 2.6
HPH	24.7 ± 2.2^*^	54.0 ± 2.3^*^
HPH + PIO (E)	13.7 ± 2.1^*^^#^	30.5 ± 1.8^*^^#^
HPH + PIO (L)	23.0 ± 2.8^*^	52.9 ± 2.2^*^

* *p* < 0.01 vs. control group.

# *p* < 0.05 vs. HPH group.

The external diameter (ED), medial wall thickness (MT), medial cross-sectional area (MA), and total arterial cross-sectional area (TAA) of peripheral pulmonary artery were measured. Then, MT and MA% were calculated as follows: MT% = (MT/ED) × 100%; MA% = (MA/TAA) × 100%.

**Figure 2:**
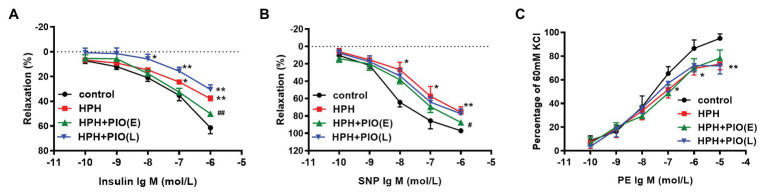
Early treatment of PIO partly reversed the system insulin resistance and the pulmonary arterial insulin resistance. **(A)** Response of the pulmonary artery of each study group to insulin. **(B)** Response of the pulmonary artery of each study group to SNP. **(C)** Response of the pulmonary artery of each study group to PE. Arterial segments of 2–5 from each rat were used. Data were presented as means ± SEM. *n* = 6. ^*^p < 0.05, ^**^p < 0.01 vs. control group; ^#^*p* < 0.05, ^##^*p* < 0.01 vs. HPH group.

Insulin has a vasodilated effect on arterial rings *in vitro*. VIR is manifested by the reduced vasodilating response to insulin (Wang et al., 2011). Insulin induced a dose-dependent vasodilation in pulmonary arteries from rats of all groups. The insulin-induced relaxation was significantly attenuated in the pulmonary arteries from HPH rats compared with the control group (Figures 2A–C), indicating VIR occurred in the pulmonary arteries in HPH rat models.

### Early Treatment of PIO Partly Alleviated Vascular Insulin Resistance and Pulmonary Hypertension

Considering PIO is a potent insulin-sensitizing agent, we next treated the HPH rats with PIO. After exposure to chronic hypoxia for 4 weeks, treatment with PIO at early stage of HPH significantly reduced mPAP and RVI (Figure 1; *p* < 0.05). However, treatment with PIO at late stage did not improve the HPH as no significant differences were found in these parameters between the HPH + PIO (L) and HPH groups. Consistently, the histopathological alterations were alleviated after early treatment of PIO in the HPH + PIO (E) group. Early treatment with PIO attenuated hypoxia-induced increases in both BNP and β-MHC levels (Figures 1F,G). However, PIO treatment in the late stage failed to reverse the histopathological alterations in the pulmonary arteries of rats in the HPH + PIO (L) group.

We next checked the insulin’s effect on pulmonary arterial rings *in vitro*. Early treatment with PIO improved insulin-induced pulmonary arterial relaxation in the HPH rats. While the insulin-induced relaxation of pulmonary arterial rings from rats in the HPH + PIO (L) group was not significantly different from that in the HPH group. In addition, responses to SNP and PE were also impaired in HPH pulmonary arteries. Early treatment with PIO partially improved SNP induced vasodilation but not PE induced vasoconstriction. These data suggested that PIO treatment in the early stage partly alleviate VIR and pulmonary hypertension.

### Dysregulation of TRIB3, PPARγ, and Insulin Signaling in the Pulmonary Arteries of Rats With HPH

**Figure 3:**
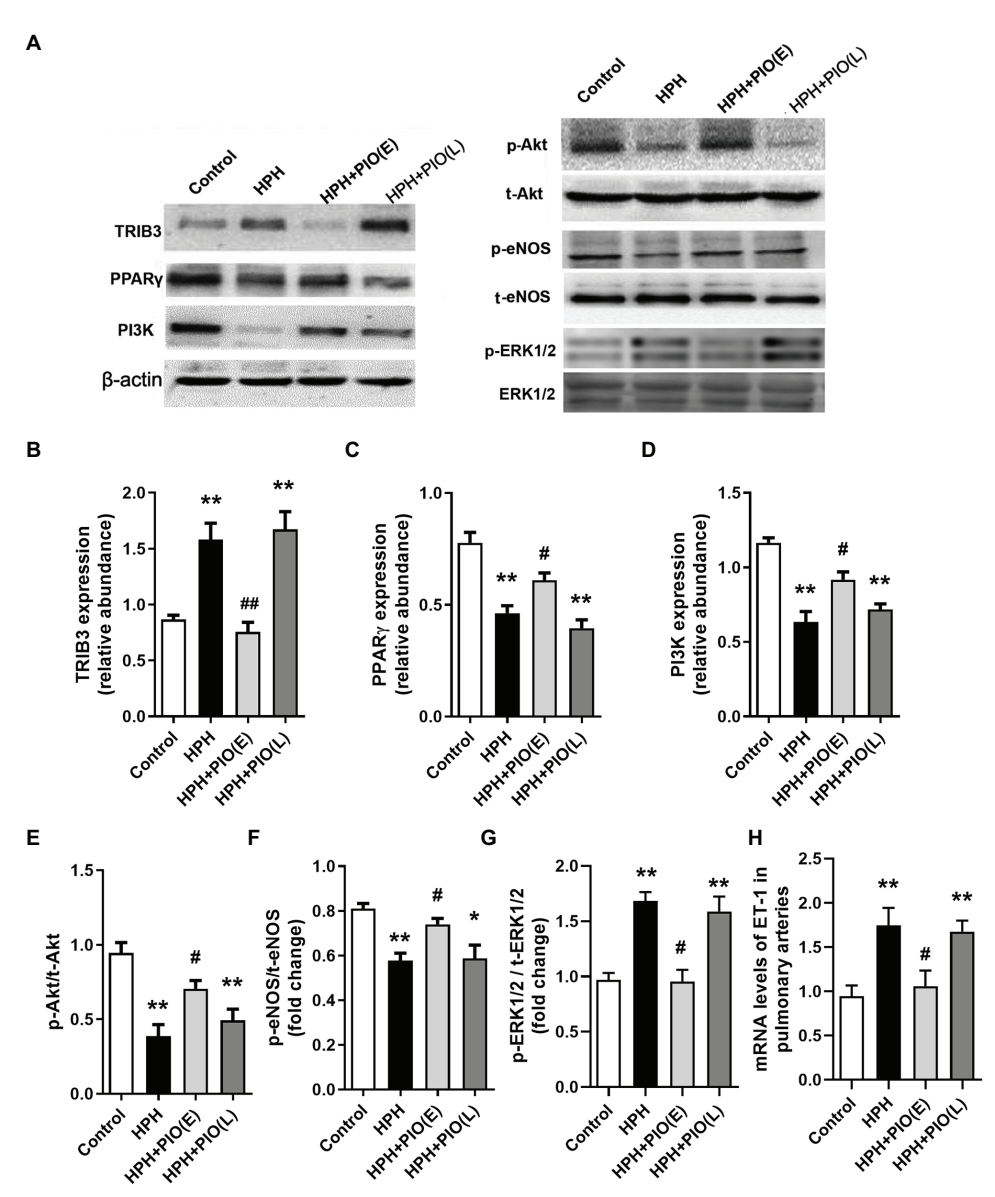
Changes in the protein levels of tribbles homolog 3 (TRIB3), PPARγ, and insulin signaling pathways in the pulmonary arteries of HPH rats. **(A)** Representative western blots showing the expressions of TRIB3, PPARγ, phosphoinositide 3-kinase (PI3K), Akt, and endothelial NO synthase (eNOS), and phosphorylations of Akt and eNOS. **(B)** Western blotting analyzing the protein TRIB3 expression. **(C)** Western blotting analyzing the protein PPARγ expression. **(D)** Western blotting analyzing the protein PI3K expression. **(E)** Western blotting analyzing ratio of phosphorylated Akt (p-Akt) to total Akt. **(F)** Western blotting analyzing ratio of phosphorylated eNOS (p-eNOS) to total eNOS. **(G)** Western blotting analyzing ratio of phosphorylated ERK1/2 (p-ERK1/2) to total ERK1/2. **(H)** mRNA levels of ET-1. Data were presented as means ± SEM. *n* = 4. ^*^p < 0.05, ^**^p < 0.01 vs. control group; ^#^*p* < 0.05, ^##^*p* < 0.01 vs. HPH group.

Tribbles homolog 3 expression significantly increased, while PPARγ expression decreased remarkably in the pulmonary arteries from HPH rat models compared with the control group. Early PIO treatment partly reversed the protein levels of both TRIB3 and PPARγ; however, no effect was noted when PIO was given in the late stage of the HPH. The levels of PI3K, p-Akt, and p-eNOS significantly reduced in the pulmonary arteries of rats with HPH compared with those in the control group. Early PIO treatment reversed the reduction of p-Akt, PI3K, and p-eNOS compared with those in the HPH group; however, late treatment failed to act in the same way (Figure 3).

### NO Production Decreased and ET-1 Production Increased in Hypoxic Primary Pulmonary Artery Endothelial Cells

**Figure 4:**
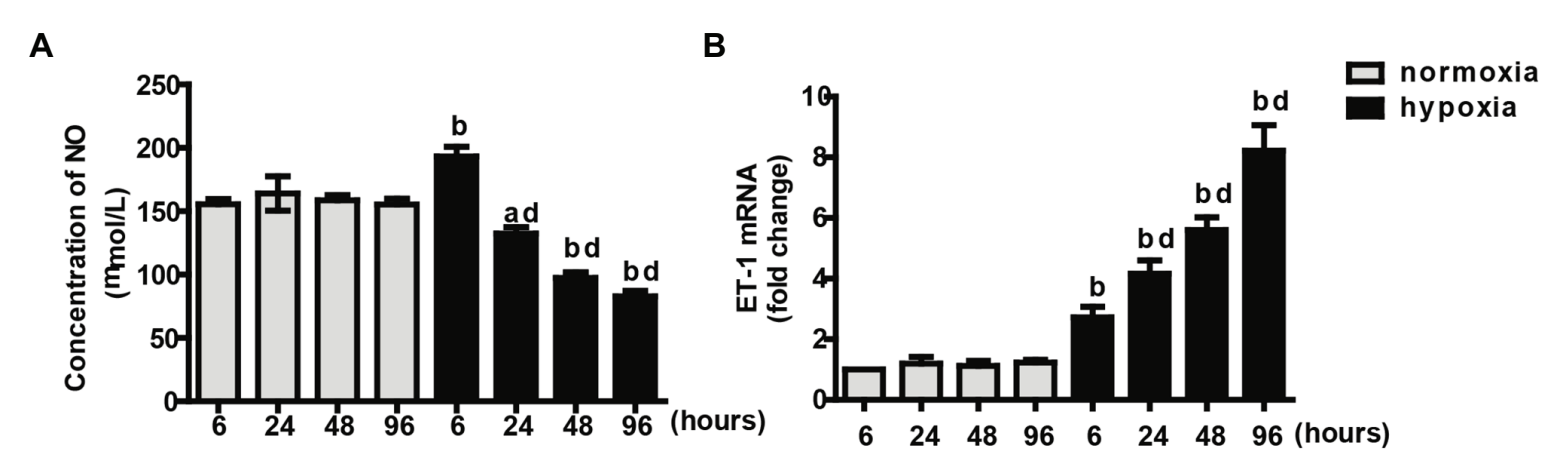
Measurements of NO concentration **(A)** and endothelin-1 (ET-1) mRNA **(B)** at different time intervals under normoxia or hypoxia condition. Values are presented as mean ± SEM, *n* = 7. ^a^*p* < 0.05, ^b^*p* < 0.01 vs. the same time point under normoxia condition; ^d^*p* < 0.01 vs. 6 h under hypoxia condition.

In the presence of insulin in the cell culture medium, the NO production by PAECs under the normoxic condition was similar for four time points. After 6 h, hypoxia induced higher NO production than that under the normoxic condition. After 24 h, the concentrations of NO under the hypoxic condition declined dramatically over time and were lower than those under the normoxic condition at each time point (Figure 4A). No significant difference in the mRNA levels of ET-1 was observed between different time intervals under the normoxic condition. ET-1 mRNA levels significantly increased under the hypoxic condition than under the normoxic condition with the same time interval; it progressively increased over time under the hypoxic condition (Figure 4B).

### Dysregulation of Proteins in PAECs in the Insulin Signaling Pathway at Different Time Intervals Under the Normoxic or Hypoxic Condition

**Figure 5:**
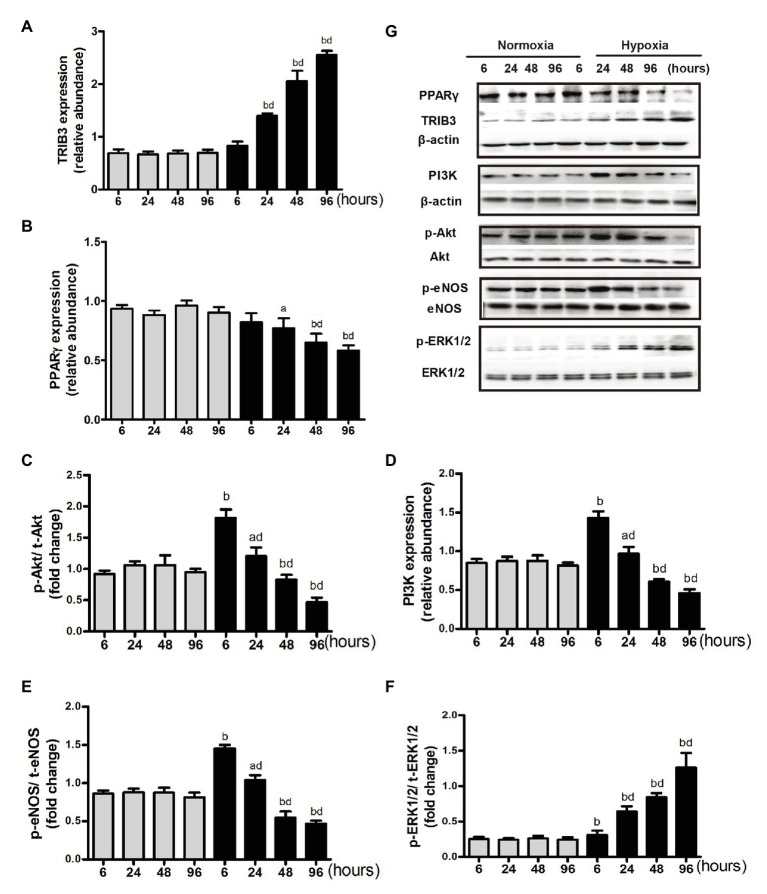
Expression of proteins in insulin signaling pathway under normoxia or hypoxia condition. **(A)** Western blotting analyzing the protein TRIB3 expression. **(B)** Western blotting analyzing the protein PPARγ expression. **(C)** Western blotting analyzing ratio of phosphorylated Akt to total Akt. **(D)** Western blotting analyzing the protein PI3K expression. **(E)** Western blotting analyzing ratio of phosphorylated eNOS to total eNOS. **(F)** Western blotting analyzing ratio of phosphorylated ERK1/2 (p-ERK1/2) to total ERK1/2. **(G)** Representative western blots showing the expressions of TRIB3, PPARγ, PI3K, Akt, eNOS, and ERK1/2, and phosphorylations of Akt, eNOS, and ERK1/2. ^a^*p* < 0.05, ^b^*p* < 0.01 vs. the same time point under normoxia condition; Values are presented as mean ± SEM, *n* = 7. ^d^*p* < 0.01 vs. 6 h under hypoxia condition.

Pulmonary arterial endothelial cells were cultured in the medium with 10^−7^ mmol/L of insulin. Among groups with different time intervals under the normoxic condition, the protein levels of TRIB3, PPARγ, PI3K, p-Akt, p-ERK1, and p-eNOS were comparable. In the acute phase of hypoxia (6 or 8 h), the protein level of TRIB3 increased significantly compared with that at the same time interval under the normoxic condition and further increased over time under the hypoxic condition. The protein levels of p-Akt, PI3K, and p-eNOS showed similar trends, which significantly increased after 6 h compared with the same time interval under the normoxic condition, and decreased over time under the hypoxic condition (Figures 5C–F). In the acute phase of hypoxia, the protein level of PPARγ was comparable with that under the normoxic condition. It decreased after 24 h under the hypoxic condition compared with the same time point under the normoxic condition, and further decreased over time (Figure 5B). The protein level of p-ERK1/2 increased at every time point under the hypoxic condition compared with the normoxic condition; it dramatically increased over time under the hypoxic condition (Figure 5).

### PPARγ and PI3K/Akt/eNOS in PAECs Were Reduced With TRIB3 Overexpression Under Normoxic and Hypoxic Conditions

**Figure 6:**
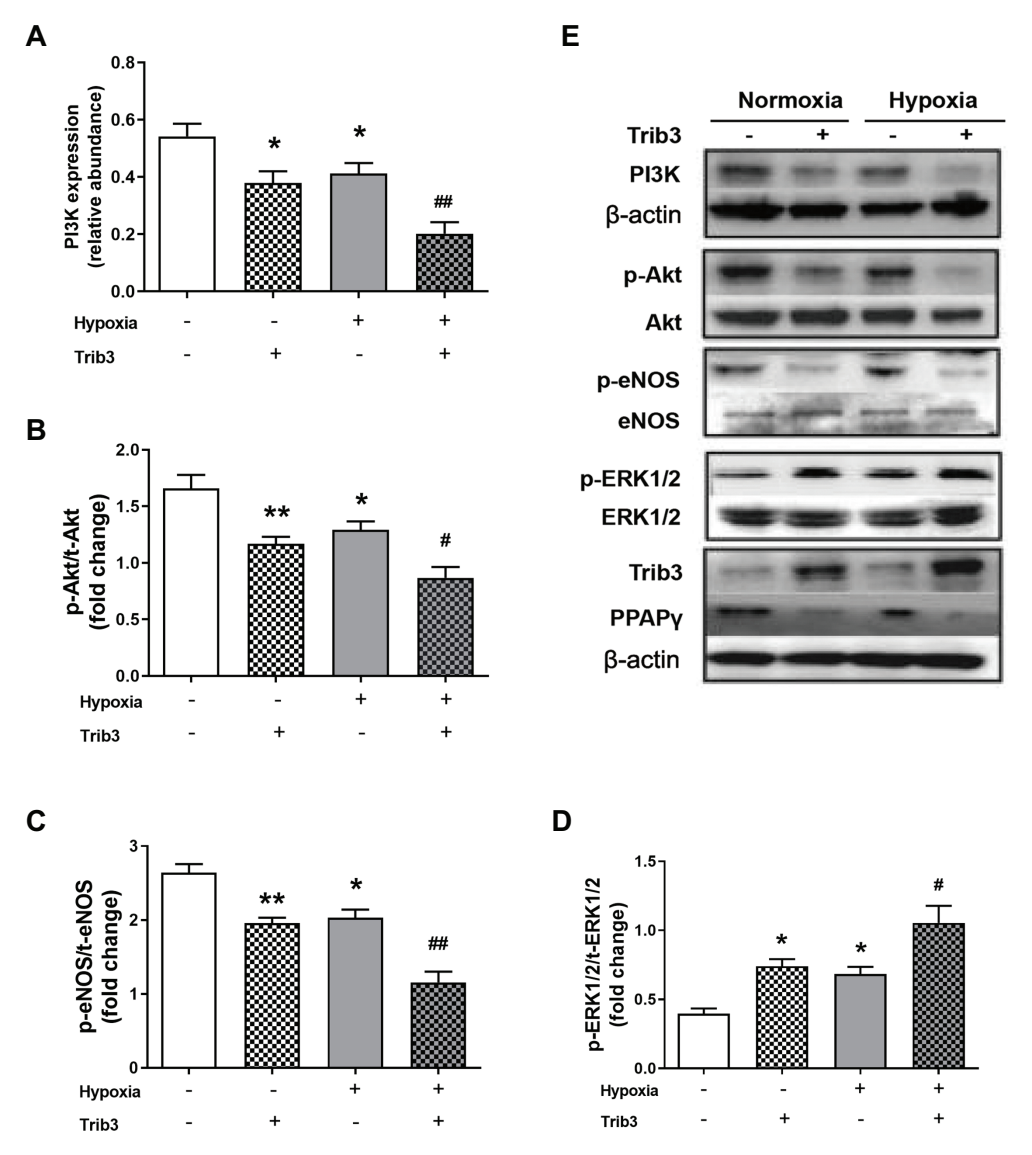
The effect of over-expression of TRIB3 with lentivirus on the protein levels of insulin signaling pathway in PAECs. **(A)** Western blotting analyzing the protein PI3K expression. **(B)** Western blotting analyzing ratio of p-Akt to total Akt. **(C)** Western blotting analyzing ratio of p-eNOS to total eNOS. **(D)** Western blotting analyzing ratio of phosphorylated extracellular signal-regulated protein kinases 1 and 2 (ERK1/2) to total ERK1/2. **(E)** Representative western blots showing the expressions of PI3K, Akt, eNOS, and ERK1/2 and phosphorylations of Akt, eNOS, and ERK1/2. Values are presented as mean ± SEM, *n* = 6. ^*^p < 0.05, ^**^p < 0.01 vs. normoxia control group; ^#^*p* < 0.05, ^##^*p* < 0.01 vs. hypoxia control group.

Pulmonary arterial endothelial cells were cultured in a medium with 10^−7^ mmol/L of insulin under hypoxic or normoxic condition, with the overexpression of TRIB3 or control vehicle. TRIB3 level was increased and PPARγ levels was decreased in PAECs under hypoxia condition. Hypoxia caused reduced PPARγ and the protein levels of PI3K, p-Akt, and p-eNOS. Importantly, overexpression of TRIB3 further decreased the protein levels of PPARγ, PI3K, p-Akt, and p-eNOS. The p-ERK1/2 levels increased in the TRIB3 + hypoxia, TRIB3 + normoxia, and control + hypoxia groups compared with the control + normoxia group, with the highest level detected in the TRIB3 + hypoxia group (Figure 6).

### TRIB3 Knockdown Improved Akt and eNOS Phosphorylations and Reduced ERK1/2 Activation in PAECs After Hypoxia

**Figure 7:**
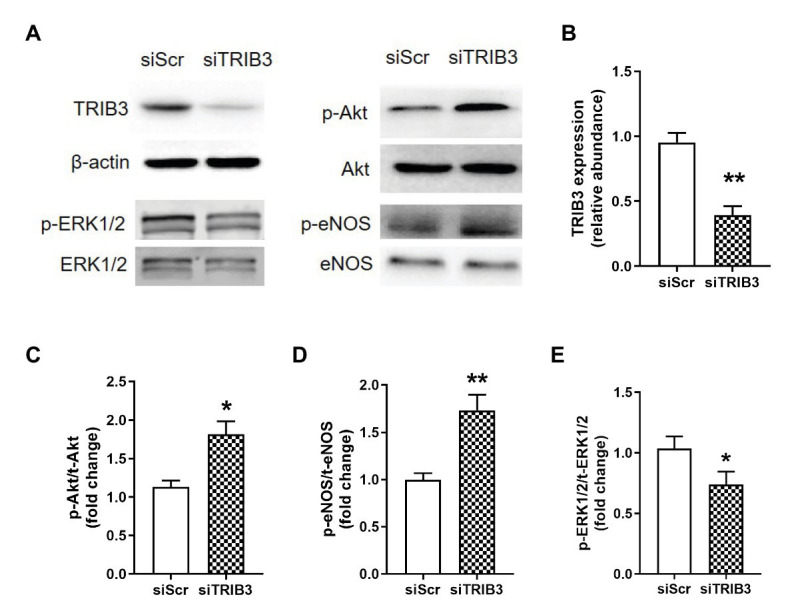
The effect of knockdown of TRIB3 with siTRIB3 or scrambled siRNA on the protein levels of insulin signaling pathway in PAECs after hypoxia. **(A)** Representative western blots showing the expressions of TRIB3, Akt, eNOS, and ERK1/2 and phosphorylations of Akt, eNOS, and ERK1/2. **(B)** Western blotting analyzing the protein TRIB3 expression. **(C)** Western blotting analyzing ratio of p-Akt to total Akt. **(D)** Western blotting analyzing ratio of p-eNOS to total eNOS. **(E)** Western blotting analyzing ratio of phosphorylated ERK1/2 to total ERK1/2. Values are presented as mean ± SEM, *n* = 4. ^*^p < 0.05, ^**^p < 0.01 vs. siScr control group.

To determine whether TRIB3 is responsible for the impaired insulin signaling, PAECs were cultured in a medium with 10^−7^ mmol/L of insulin under hypoxic condition and measured the activation of insulin signaling with TRIB3 knockdown by siRNA. The results showed that TRIB3 was significantly reduced by siRNA in PAECs. The effect of knockdown of TRIB3 with siTRIB3 or scrambled siRNA on insulin signaling pathway in hypoxia treated PAECs was detected by western blot. p-Akt and p-eNOS were significantly enhanced in the PAECs after knockdown of TRIB3, while activation of ERK1/2 was markedly decreased after siTRIB3 treatment (Figure 7).

## Discussion

The present study has novel findings that VIR mediated by TRIB3 played an important role in the development of HPH. And improvement of the vascular insulin sensitivity at early stage by PIO alleviated HPH.

Insulin’s actions on vascular are of great importance as it enhances the compliance of arteries, relaxes arterioles to increase tissue blood flow, and expands perfusion. The present study reveals that VIR is closely associated with the development of HPH. The disturbance of the balance between ET-1 and NO weakened the vasodilating activity and increased the vasoconstrictive activity of the vessels. Disturbance of endothelium may further lead to vessel injury, remodeling, thrombogenesis, and occlusion. We also observed increased systemic blood glucose and insulin levels in HPH rats. Research has shown that hypoxia acutely or chronically influences the control of blood glucose and related hormones. In our study, the increase of glucose is likely due to the intermittent nature of the hypoxia challenge. This is a limitation of the study.

The present study showed an association between hypoxia-induced TRIB3 expression and VIR in pulmonary arteries. TRIB3 levels were increased in pulmonary arteries from HPH rats, accompanied with reduced PPARγ, and imbalance between insulin-induced Akt/eNOS/NO and ERK1/2/ET-1 signaling. Overexpression of TRIB3 in PAECs caused impaired Akt and eNOS activation. These findings were consistent with previous observations that pathological overexpression of TRIB3 blocked insulin-induced Akt phosphorylation, negatively regulated insulin action, and contributed to hyperglycemia and insulin resistance in liver and skeletal muscle (Du et al., 2003; Liu et al., 2010). Therefore, upregulated TRIB3 decreased the level of p-Akt and distorted subsequent signaling pathways. On the other hand, overexpression of TRIB3 in PAECs enhanced insulin-stimulated ERK1/2 phosphorylation. TRIB3 is a known regulator of MAPK–ERK pathway in cancer and cardiomyopathy models (Tang et al., 2008; Izrailit et al., 2013) by directly interacted with ERK or increased posttranslational phosphorylation. Moreover, TRIB3 knockdown caused significant improvement in Akt and eNOS phosphorylations and otherwise a reduction of ERK1/2 activation in PAECs after hypoxia, suggesting that TRIB3 mediates hypoxia-induced VIR and endothelial dysfunction.

Most recent study found that PPARγ activation by PIO prevents pulmonary arterial hypertension, the pathogenesis of which is different from that HPH. The present study for the first time identified PIO treatment at the early stage benefits hypoxia-induced pulmonary arterial dysfunction and HPH, which is at least partially relies on the improvement of vascular insulin sensitivity and vasodilation. TRIB3 has been identified acting as a potent negative regulator of PPARγ (Takahashi et al., 2008), indicating that the increase of TRIB3 level may be responsible for the limit effects of late PIO treatment.

The present study provided new insights into the development of therapeutics for HPH. Hypoxia-induced TRIB3 upregulation is a potential molecular basis of HPH. TRIB3 suppresses PPARγ and regulates insulin signaling pathway, resulting in the reduction of vascular NO production and raise of ET-1 secretion. Nevertheless, whether other subtypes of PH can benefit from improving insulin sensitivity or TRIB3 reduction need further study. TRIB3 is also a positive regulator of canonical TGF-β signaling to regulate fibroblast activation and tissue fibrosis (Tomcik et al., 2016). PPARγ, which could be regulated by TRIB3, was reported that inhibited TGF-β signaling and acted as a link between pro-proliferative TGF-β and anti-proliferative bone morphogenetic protein 2 (BMP2) signalings in vascular smooth muscle cells and inhibited pulmonary arterial hypertension (Calvier et al., 2017). Additionally, TRIB3 deficiency ameliorates metabolic disturbance inflammation, fibrosis, and myocardial hypertrophy in rats with dilated cardiomyopathy (Ti et al., 2011) indicating the potential benefits of TRIB3 regarding its anti-fibrotic and metabolic modulating effects.

In summary, this study indicated that VIR plays an important role in the development of HPH. The impaired insulin-induced pulmonary vasodilation and the imbalance of insulin-induced signalings are mediated by TRIB3 upregulation in the endothelium of HPH. PIO treatment beginning at the early stage improves vascular insulin sensitivity that may help to limit the progression of hypoxic pulmonary hypertension, which could provide a new perspective in managing patients with HPH, focusing on improving the pulmonary vascular IR in the early stage.

## Data Availability Statement

All datasets generated for this study are included in the article/supplementary material.

## Ethics Statement

The animal study was reviewed and approved by the ethics committee of the Animal Experimentation of the Fourth Military Medical University.

## Author Contributions

FF, JH, and HS carried out the studies, collected data, and drafted the manuscript. HW, QD, and MZ collected data and performed the statistical analysis. HZ, WX, and XS designed the study and revised the manuscript. All authors contributed to the article and approved the submitted version.

### Conflict of Interest

The authors declare that the research was conducted in the absence of any commercial or financial relationships that could be construed as a potential conflict of interest.
